# Anticoagulant regimens for different therapeutic plasma exchange modes

**DOI:** 10.3389/fmed.2025.1568333

**Published:** 2025-06-18

**Authors:** Ruishan Yao, Haiyan Huang, Yaohui Ming, Huiling Guo, Lingli Miao, Su Wang, Jia Wan, Jin Zhou, Xiaofang Wang, Yanli Wu, Yaowen Yuan, Ting Wang, Ruiting Li

**Affiliations:** Department of Critical Care Medicine, Union Hospital, Tongji Medical College, Huazhong University of Science and Technology, Wuhan, China

**Keywords:** therapeutic plasma exchange, centrifugal TPE, membrane TPE, anticoagulant therapy, anticoagulant drug

## Abstract

Therapeutic plasma exchange (TPE) is widely used in various fields of clinical practice. There are two main modes of TPE: centrifugal TPE (cTPE) and membrane TPE (mTPE). No matter which mode of TPE, anticoagulant therapy is essential to prevent clotting in the extracorporeal circuit. Therefore, selecting a suitable and safe anticoagulant is crucial. Currently, heparin and citrate are the most commonly used anticoagulants. Different anticoagulant regimens have varying advantages, disadvantages, and associated complications, making them suitable for different TPE modes. This review aims to summarize relevant research findings to provide clinicians with evidence to guide their selection of appropriate anticoagulants during TPE.

## 1 Introduction

Therapeutic plasma exchange (TPE) has been extensively applied in various fields, including hepatology, nephrology, hematology, neurology, immunology, and organ transplantation (1, 2). In clinical practice, there are two modes of TPE: centrifugal TPE (cTPE) and membrane TPE (mTPE) (3). Globally, the selection of TPE modes hinges on local economics and preferences (4). It has been reported that mTPE is prevalently utilized in China, Japan, and Germany, while cTPE is widely used in the United States (5, 6).

Anticoagulant therapy is important to prevent extracorporeal circuit clotting irrespective of the TPE mode. Frequent clotting in the circuit not only reduces the efficacy of treatment but also escalates the treatment cost and exacerbates blood loss. Conversely, excessive anticoagulation may lead to bleeding, posing a potential threat to the patients’ lives. Hence, it is imperative to choose a suitable and safe anticoagulant. Currently, heparin and citrate are the most commonly used anticoagulants. Nevertheless, the selection of anticoagulants for different TPE modes has been controversial (7–10). This study endeavors to summarize relevant research findings, aiming to offer evidence for clinicians to select appropriate anticoagulants during TPE.

## 2 Importance of anticoagulant therapy and coagulation status evaluation

### 2.1 Anticoagulant therapy

TPE involves separating the plasma of patients from whole blood using plasma separation technology. Subsequently, the plasma or plasma substitute products are replenished to clear pathogenic factors, such as autoantibodies, immune complexes, lipoproteins, and toxins in the plasma, mitigating the adverse effects of these factors and alleviating pathological processes (11). The blood clotting mechanism is activated once the blood is drawn. To ensure the smooth progress of treatment, anticoagulation therapy is recommended. Some studies have reported that at most treatment centers, TPE is performed without anticoagulation, and the incidence of tube clotting is 2.1%. Thus, anticoagulation therapy was considered non-mandatory. However, this recommendation was based on single-center work experience (12). Scholars from various countries recognize the importance of anticoagulants during TPE (3–11). Yuan et al. (7) reported a high clotting frequency in the filter in the non-anticoagulant group (33%), which increased the treatment duration and cost, and also contributed to the failure of treatment completion and the need to replace the pipeline. Procedural delay can have adverse effects on the plasma because it has a limited shelf life. Moreover, pipeline clotting can lead to anemia or thrombocytopenia in patients. Therefore, in clinical practice, anticoagulants are used for TPE. Failing to use anticoagulants cannot guarantee clinical therapeutic effects, can cause physical, mental, and economic damage to patients, and can increase the burden of nursing work.

### 2.2 Evaluation of the blood coagulation status

The use of anticoagulants raises the risk of bleeding. Therefore, patients need to be evaluated for bleeding and clotting status before TPE. This evaluation includes inquiring about patients’ past medical histories, assessing the risk of hemorrhagic and thromboembolic diseases, and evaluating the coagulation indices of patients (13). Evaluating coagulation status can assist clinicians in selecting the appropriate anticoagulation method and monitoring the effect of anticoagulants. For example, activated partial thromboplastin time (APTT), activated clotting time (ACT), prothrombin time (PT), thrombin time (TT), fibrinogen degradation products (FDP), fibrinogen and thromboela-stogram (TEG) are used to detect potential bleeding tendencies, while platelet count is used to determine platelet function. Consequently, clinicians must select appropriate anticoagulants and administer precise anticoagulant dosages according to patients’ coagulation function, bleeding risk, as well as the indications and contraindications.

## 3 Anticoagulant drugs, dosage, and monitoring

### 3.1 Systemic anticoagulation

#### 3.1.1 Unfractionated heparin

UFH [molecular weight: 5,000–30,000 Dalton (Da)] is a commonly used anticoagulant in blood purification therapy. The anticoagulant effect of UFH can be attributed to the upregulation of antithrombin III (ATIII) activity and the inhibition of thrombin (IIA and Xa) activity, prolonging APTT (13–18). Systemic anticoagulant effects can be achieved in 3–5 min post-anticoagulant administration. Heparin is mainly metabolized by the liver and reticuloendothelial system (16). The physiological half-life of UFH is 30 min, which can be extended to 3 h in patients with renal failure (16, 17). At the beginning of TPE, patients are administered with UFH at a dose of 62.5–125 U/kg (0.5–1 mg/kg), and subsequently, UFH is administered at a dose of 1,250–2,500 U/h (10–20 mg/h) as needed. Dosing is stopped 30 min before the end of treatment (11). APTT is a reliable indicator of the anticoagulant effect and safety of heparin. The APTT value should be maintained at levels that are 1.5–2.5 times the physiological value to achieve anticoagulant effects and mitigate bleeding risk (14, 18). The advantages of UFH are its cost-effectiveness, ease of use, ease of monitoring the anticoagulant effect, and ability to be neutralized using protamine. The disadvantages of UFH include the unpredictable pharmacokinetics resulting in dosing variability, heparin resistance due to low antithrombin levels, the development of heparin-induced thrombocytopenia (HIT), and an increased risk of haemorrhage (13, 14). Previous studies have reported that the incidence rate of heparin-induced bleeding is 10–50%, which is caused by prolonged APTT. Therefore, heparin is contraindicated in patients who are bleeding or at high risk of bleeding due to recent surgery, trauma, and coagulation dysfunction, as well as in patients with HIT (Table 1).

**TABLE 1:** Comparison of two common anticoagulant drugs.

	Unfractionated heparin	Citrate
Component	Mucopolysaccharide sulfate	Sodium citrate (weak acid)
Molecular weight	5,000–100,000 Da	294 Da
Main anticoagulation mechanism	Enhancing activity of ATIII, inhibiting activity of clotting factors (IIa and Xa), prolonging APTT	Anticoagulation is achieved by chelating Ca^2+^
Application situation	Low bleeding risk; mTPE	No citrate anticoagulant contraindications; cTPE
Onset time	3–5 min	Immediately
Metabolism	Liver metabolism, renal excretion; normal half-life: 0.5 h; renal failure: 3 h	Substrate of tricarboxylic acid cycle; metabolized by aerobic metabolism in liver, kidney, and skeletal muscle; metabolic time: 5 min
Elimination by CRRT	No	Partially removed
Dosage	Initial: 62.5–125 U/kg (0.5–1 mg/kg); maintenance: 1,250–2,500 U/h (10–20 mg/h)	Initial dose is adjusted by blood flow, and then adjusted according to monitoring results
Monitoring index	APTT every 4–6 h (1.5–2.5 times); every 12 h after stabilization	Every 0.5–2 h (× 4) -every 4 h (× 4)- every 6–8 h after stabilization; total calcium and blood magnesium were monitored every 24 h; pre-filter Ca^2+^: 1.0–1.2 mmol/l; post-filter Ca^2+^: 0.25–0.45 mmol/l; total calcium: 2.25–2.75 mmol/l
Antagonist	Protamine	Calcium
Complications	Bleeding, HIT	Metabolic acidosis, metabolic alkalosis; hypocalcemia, hypercalcemia, hypomagnesemia, hypernatremia

ATIII, antithrombin III; cTPE, centrifugal TPE; mTPE, membrane TPE; HIT, heparin-induced thrombocytopenia; APTT, activated partial thromboplastin time.

#### 3.1.2 Low-molecular-weight heparin

LMWH (molecular weight: approximately 4,000–6,000 Da) is produced through the chemical degradation or enzymatic hydrolysis of ordinary heparin (13, 15). LMWH exerts anticoagulant effects mainly by inhibiting Xa activation (13–18). The prolonged half-life of LMWH (2–4 h) is due to its clearance by the kidney (13, 14, 18). Patients with renal insufficiency and undergoing dialysis exhibit delayed LMWH metabolism. The excessive use of LMWH increases the risk of bleeding. In TPE, LMWH is intravenously administered at a dose of 60–80 IU/kg body weight 20–30 min before treatment without additional dosing. The anticoagulation effect of LMWH is monitored based on the anti-Xa levels with target levels being 0.25–0.35 IU/mL(14, 18). Compared with UFH, LMWH has the advantages of simple pharmacokinetics and reliable anticoagulant response. The disadvantages of LMWH include prolonged anticoagulation effects in patients with renal failure and poor reversal with protamine. Additionally, the need to test anti-Xa activity increases the monitoring cost. Similar to UFH, LMWH is contraindicated in patients at a high risk of bleeding due to its systemic effects (13, 14, 18).

#### 3.1.3 Thrombin antagonists

Thrombin inhibitors (argatroban and bivalirudin) are used as an alternative to UFH in patients with HIT. Argatroban, a commonly used thrombin inhibitor in patients with HIT (14), is a synthetic small-molecule drug (molecular weight: 527 Da). Additionally, argatroban can reversibly bind to the active site of thrombin and inhibit thrombin-catalyzed reactions, including the formation of fibrin, the activation of coagulation factors V, VIII, and PC, and platelet aggregation (13). Argatroban is primarily metabolized by the liver (13, 14, 18, 19). The half-life of argatroban in individuals with physiological renal function is 15–30 min (13, 14, 18, 19). During TPE, patients are administered argatroban at a dose of 0.05–0.1 mg/kg and subsequently at a dose of 1–3 mg/h as needed. Dosing is stopped 20–30 min before the end of treatment.

Bivalirudin can be used as an alternative to argatroban in patients with liver and kidney failure. Bivalirudin has a short half-life, exhibits reversible thrombin binding, and is cleared via extra-renal and extra-hepatic mechanisms (20). The pharmacological advantages of bivalirudin, a hirudin analog, include decreased dependence on renal clearance and a low allergic reaction rate. The target APTT level is 1.5–2.0 times the physiological level of APTT (13, 19).

#### 3.1.4 Nafamostat mesilate (NM)

NM, a serine protease inhibitor (molecular weight: 540 Da), mainly inhibits thrombin, Factor XIa, Xa, XII, the kallikrein-kinin system, the fibrinolysis system, the complement, and platelet activation (13). The liver and blood are the main sites for NM metabolism. The half-life of NM is 5–8 min, which has little effect on blood coagulation function *in vivo*. Thus, NM is a novel anticoagulant used in cardiopulmonary bypass, especially for patients at risk of bleeding or with active bleeding (13, 21). During TPE, 40 mg of NM in 1,000 mL saline is used for pipeline precharging. NM is administered at an initial dosage of 20–50 mg/h for continuous intravenous infusion (22). It is recommended to monitor the ACT or the APTT to guide the adjustment of NM dose. The dose is adjusted according to the risk of blood clotting, the anticoagulant effect, and the blood clotting function (22). Nakamura et al. (23) reported that NM exhibited good safety and efficacy profiles as an anticoagulant in TPE for high-risk bleeding populations. However, NM is associated with several side effects and is expensive (24, 25), which limits its clinical application.

### 3.2 Regional anticoagulation

#### 3.2.1 Regional citrate anticoagulation (RCA)

The relative molecular weight of sodium citrate (citrate) is 294 Da. RCA is a special mode of *in vitro* anticoagulation therapy. The basic principle of RCA involves the injection of citrate into the extraction end of circulation loop. Citrate reversibly chelates ionized calcium (iCa) in the circuit. As a result, the coagulation mechanism is inhibited when the concentration of iCa in the circulation loop is less than 0.45 mmol/L. Previous studies have demonstrated that regulating *in vitro* iCa concentrations at 0.25–0.45 mmol/L can achieve the anticoagulation effect of the circulation loop (13, 14, 18, 26). However, an equal amount of cleared iCa should be added at the end of the circulation loop to ensure that the *in vivo* iCa level is 1.0–1.2 mmol/L (27, 28) (Table 1).

### 3.3 Non-anticoagulation therapy

Performing TPE without anticoagulants is a controversial approach. Currently, non-anticoagulant therapy is recommended for patients at high risk of bleeding and those with contraindications to citrate. Previous studies have reported that the incidence of clotting in the TPE extracorporeal circulation in the non-anticoagulation mode ranges from 6.3 to 33% (6, 7, 9). The incidence of clotting in non-coagulation therapy varies. Even when the non-anticoagulant mode does not increase the bleeding risk, the clotting system can be activated in some patients with high-risk bleeding and active bleeding. Additionally, the risk of clotting in the extracorporeal circulation pipeline increases significantly, which adversely affects the treatment efficiency and increases the medical burden on patients. However, non-anticoagulant mode is the preferred option for TPE in patients with contraindications to anticoagulants.

## 4 Selection of anticoagulants for different TPE modes

Heparin and citrate are the most commonly used anticoagulants. For patients with contraindications for both citrate and heparin, argatroban and NM can be used to prevent coagulation in the extracorporeal loop (29). Previously, citrate and heparin were commonly used for cTPE and mTPE, respectively. However, citrate has also been recently used for mTPE (8–10), especially for patients at high risk of bleeding. According to the World Apheresis Registry (WAR) (12), TPE is the most commonly used treatment for therapeutic apheresis, with cTPE and mTPE accounting for 34.8 and 0.8% of treatments, respectively. The most commonly used anticoagulation method is RCA.

### 4.1 Selection of anticoagulant for mTPE

**FIGURE 1:**
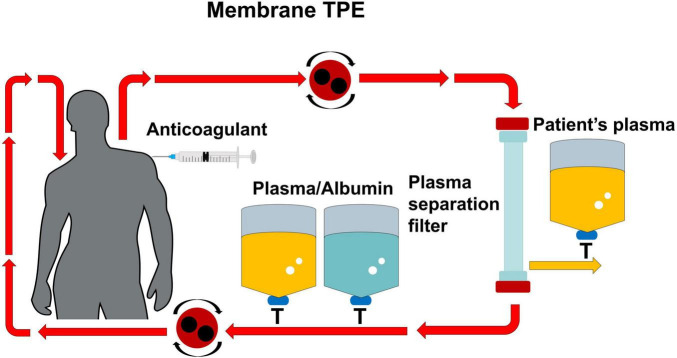
Anticoagulation regimen for membrane TPE. In membrane TPE, the patient’ plasma is separated through a hollow fiber filter. HA is used as the anticoagulation method. The replacement fluid (e.g., fresh frozen plasma or albumin) is infused post-filtration before returning to the patient. Membrane TPE, membrane therapeutic plasma exchange; HA, heparin anticoagulation.

Heparin anticoagulation (HA) is the most widely used method in mTPE and has the advantages of satisfactory anticoagulation effect, rapid onset, and low cost (7). In mTPE, approximately 70% of citrate enters the body because of the low filter clearance rate of citrate, which increases the metabolic burden and may induce complications such as metabolic alkalosis and hypocalcemia. Hence, several hemodialysis centers use HA as the standard anticoagulation procedure for mTPE (29) (Figure 1). However, clinical studies have reported significant side effects of HA(30). Heparin cannot be used in patients with HIT or severe bleeding. Therefore, researchers advocate the extensive application of RCA in mTPE. Recently, several hospitals have adopted RCA as the preferred anticoagulation method for mTPE (7, 31). The dose of citrate pumped into patients is directly correlated with its effects. Thus, several studies have focused on examining the minimum citrate required to meet the anticoagulant effect. TPE requires large infusions of fresh frozen plasma (FFP), the concentration of citrate in FFP ranges from 17 to 21 mmol/L (32). Therefore, compared to continuous renal replacement therapy (CRRT), TPE has a lower citrate demand. Yuan et al. (7) reported that during mTPE, the initial pumping rate of 4% citrate is set at 1.8% of the blood flow rate can effectively exert anticoagulation effects. Patients did not develop citrate-related side effects and electrolyte disturbances because of careful patient monitoring and intravenous calcium supplementation. This suggests that low-dose RCA is an effective anticoagulation method for mTPE. Additionally, calcium infusion should be provided at the end of the circuit of mTPE to maintain the physiological iCa level *in vivo*. Yuan et al. (7) suggested the initiation of 10% calcium gluconate at a rate of 25–30 mL/h with a monitoring interval of once every 30 min. If the parameters are stable, the monitoring interval can be extended appropriately. Thus, clinical studies have reported that the routine use of low-dose RCA during mTPE is safe and effective and can be widely used in clinical practice.

### 4.2 Selection of anticoagulant for cTPE

**FIGURE 2:**
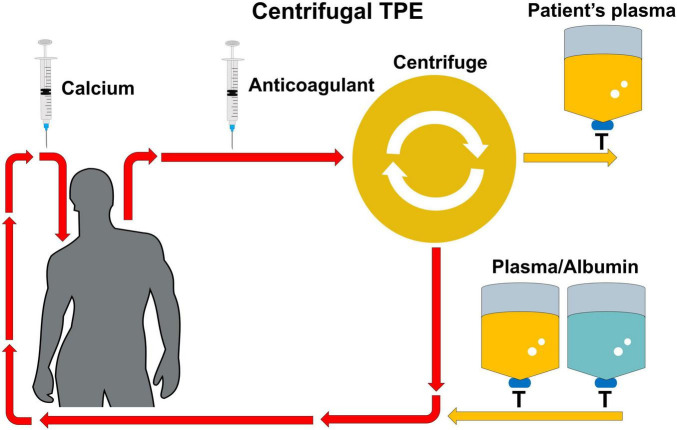
Anticoagulation regimen for centrifugal TPE. In centrifugal TPE, blood is withdrawn from the patient, and RCA is used as the anticoagulation method. The blood undergoes centrifugation to separate plasma, which is then replaced with a replacement fluid (e.g., fresh frozen plasma or albumin) before being reinfused into the patient. Centrifugal TPE, centrifugal therapeutic plasma exchange; RCA, regional citrate anticoagulation.

Clinical studies have recommended that RCA is the optimal anticoagulation method for cTPE (8, 33). The clearance rate of citrate in cTPE can reach up to 80%. Hence, the incidence of citrate-induced toxic reactions and metabolic alkalosis is markedly mitigated (33). When citrate is used as an anticoagulant during cTPE, the ratio of the 4% citrate rate to the blood flow rate is set at 1.2:1, which can ensure effective anticoagulation in the cTPE pipeline (34) (Figure 2). However, the blood iCa concentration is markedly decreased because of the high demand for citrate and the high iCa clearance rate in cTPE. In patients using FFP as a replacement fluid, the citrate in the plasma further binds to the iCa in the blood. Therefore, continuous calcium supplementation is needed to prevent hypocalcemia during cTPE. Coirier et al. (33) revealed that infusing 10% calcium gluconate at a rate of 20 mL/h during cTPE, along with the anticoagulant effect of citrate, can prevent the occurrence of hypocalcemia.

## 5 Complications related to the use of TPE anticoagulants

### 5.1 Bleeding

Bleeding is one of the most common complications of anticoagulation therapy, especially when HA is used (35). HA exerts a systemic anticoagulant effect by prolonging APTT and increases the risk of bleeding. Previous studies have confirmed HA increases the risk of bleeding by 50% with every 10 s increase in APTT (36). Thus, heparin should not be administered to patients with heparin-induced HIT or AT-III deficiency (35–37). Weiss et al. (38) reported that citrate affected the activation and adhesion of platelets and white blood cells in a concentration-dependent manner, exerting anticoagulant effects. However, the low-dose infusion of citrate has enabled the maintenance of low concentrations of citrate in patients, minimally affecting the patients’ coagulation system and preventing bleeding complications (7). Several studies have demonstrated that RCA-TPE is associated with a low risk of bleeding (7, 39, 40). Jiao et al. (35) performed a multivariate analysis of bleeding events and identified HA as one of the independent risk factors for the occurrence of bleeding events in mTPE. Additionally, the authors revealed that the bleeding event in the HA-mTPE mode was 2.666 times higher than that in the RCA-mTPE mode (35). Therefore, the coagulation index and systemic bleeding status of patients should be carefully monitored, and the heparin dosage should be adjusted accordingly in HA-TPE mode. The use of HA in patients at a high risk of bleeding undergoing TPE is controversial (Table 2). If excessive anticoagulant use is detected, we must prepare the corresponding antagonists: for heparin and LMWH, protamine can be used as an antidote, while for citrate anticoagulants, increasing calcium infusion can help prevent bleeding caused by hypocalcemia.

**TABLE 2:** Monitoring and management of complications related to TPE anticoagulation.

Complication	Bleeding	Metabolic alkalosis	Citrate accumulation	Hypocalcemia	Hypernatronemia	Hypomagnesemia
Anticoagulation regimen prone to occurrence	HA	RCA	RCA	RCA	RCA	RCA
Monitoring method	Coagulation function; TEG	Blood gas analysis	Blood gas analysis; blood biochemical indexes	Blood gas analysis	Blood gas analysis	Blood biochemical indexes
Evaluation criterion	Ecchymosis; gastrointestinal hemorrhage; cerebral hemorrhage; pulmonary hemorrhage; and other symptoms	PH > 7.45; HCO3^–^ > 27 mmol/L	Serum Ca^2+^ level; TCa/iCa ratio; acid-base state; anion gap	Serum Ca^2+^ level	Serum Na^+^ level	Serum Mg^2+^ level
Severity	Severe	Correctable	Severe	Correctable	Correctable	Correctable
Probability of occurrence	High	High	Low	High	Low	Low
Management	Protamine neutralization	Correcting metabolic alkalosis; in severe cases, CRRT	Replace the anticoagulant regimen	Calcium supplementation	Dynamic observation in severe cases, CRRT	Magnesium ion supplementation

RCA, Regional citrate anticoagulation; HA, Heparin anticoagulation; TEG, Thromboelastogram; CRRT, continuous renal replacement therapy.

### 5.2 HIT

UFH and LMWH can induce thrombocytopenia. A recent meta-analysis revealed that the risk rates of HIT with the usage of UFH and LMWH were 2.6 and 0.2%, respectively (41). Type I HIT is a non-immunogenic reaction that occurs 1–2 days after heparin administration. Patients with type I HIT exhibit a slight decrease in the platelet count without thrombus formation or bleeding, and this condition can be alleviated without stopping the drug. Type II HIT is an immunoreactive thrombocytopenia mediated by the combination of heparin and platelet factor IV to form a complex (PF4-H), which stimulates the production of IgG antibodies and activates platelets and the clotting pathway. Type II HIT occurs 5–10 days after heparin administration but also can occur within 24 h–3 weeks. Most cases of type II HIT are characterized by platelet reduction by more than 50% relative to the baseline value and can be accompanied by severe thromboembolism and acute systemic reactions (18, 41). Regular heparin and LMWH should be discontinued immediately upon diagnosis of HIT. These patients must be administered with argatroban or NM as anticoagulants.

### 5.3 Filter and pipeline coagulation

Filter and pipeline clotting, which is common in mTPE, occurs when the dose of anticoagulant is insufficient. Previous studies have reported that the average coagulation incidence in the HA-mTPE extracorporeal circulation pipeline is 1.8–7.5% (7, 9, 39, 42). Meanwhile, the incidence of coagulation in the RCA group is 0–4.4% (35). Patients with complex and changeable conditions and individual differences may have a high risk of bleeding along with blood hypercoagulability. Conventional doses of anticoagulants may not reach the effective concentration for anticoagulation, increasing the risk of blood clotting in the circulation loop. Therefore, before treatment, the patient’s condition, relevant coagulation indicators, and appropriate anticoagulant selection should be considered. The anticoagulant dose should be increased for patients with blood hypercoagulability and hyperlipidemia. Additionally, sufficient anticoagulant drugs should be prepared to avoid interruption of anticoagulant therapy. Moreover, during treatment, the coagulation status in the filters and pipeline must be monitored. When clotting occurs, the dosage of anticoagulant drugs can be appropriately increased to prevent unnecessary clotting events in the filters and pipeline.

### 5.4 Metabolic alkalosis

The concentration of citrate in the FFP is reported to be 17–21 mmol/L (32). RCA-TPE will additionally increase citrate concentration. In mTPE, 30% of the citrate is removed by the filter, while in cTPE, 80% of the citrate is removed by the filter (11). Citrate that is not removed during TPE enters the body and is metabolized to produce a large amount of bicarbonate. Unlike CRRT, TPE does not use an acidic replacement solution to neutralize the bicarbonate produced through citrate metabolism (7). Therefore, when citrate is used in TPE, the occurrence of metabolic alkalosis, especially in the mTPE mode, must be monitored. Metabolic alkalosis can occur during RCA-mTPE (7, 39, 40). Shunkwiler et al. (43) confirmed that RCA-mTPE was associated with a higher incidence of metabolic alkalosis than HA-mTPE. Metabolic alkalosis is likely to occur during continuous RCA-mTPE (35). The occurrence of metabolic alkalosis in RCA-cTPE has not been previously reported, which can be due to the limited number of clinical studies on cTPE, the high clearance rate of citrate in cTPE, and the low production of bicarbonate. However, further studies are needed to investigate the occurrence of metabolic alkalosis during RCA-cTPE. Therefore, the acid-base balance of patients undergoing RCA-TPE should be closely monitored to prevent the occurrence of metabolic alkalosis. Metabolic alkalosis, a complication of citrate use, can be easily corrected (44). For patients at risk of developing metabolic alkalosis, the pumping rate of citrate can be reduced without compromising the therapeutic effect. Severe metabolic alkalosis, which is common in patients with severe renal failure, can be corrected using CRRT (45). Kaushik et al. (46) revealed that the parallel or sequential combination of TPE and CRRT is technically feasible and safe for patients undergoing TPE and requiring hemodialysis at the same time and can effectively alleviate citrate-induced metabolic alkalosis (Table 2).

### 5.5 Citrate accumulation (CA)

CA is a serious complication of RCA. In patients with liver failure, severe shock, or hypoxemia, citrate that enters the body cannot be metabolized, which increases the incidence of CA (47, 48). Severe CA can lead to hypocalcemia and metabolic acidosis, which can be life-threatening for critically ill patients. The total calcium (TCa)/iCa ratio of ≥ 2.5 was used as an indirect standard to determine the occurrence of CA (48, 49). However, continuous citrate pumping in RCA-TPE increases the occurrence of hypocalcemia, raising the TCa/iCa ratio to ≥ 2.5 even without CA. The occurrence of CA should be comprehensively evaluated according to the following four criteria: serum Ca^2+^ level, TCa/iCa ratio, acid-base status, and anion gap change. These criteria have been widely used in the safety assessment of RCA-CRRT (35, 50). Based on Khadzhynov’s rigorous evaluation criteria, several studies have reported a low incidence of metabolic acidosis after RCA-mTPE (39, 40). The occurrence of CA has not been reported in patients undergoing RCA-cTPE, which may be related to the high rate of citrate clearance in the cTPE mode. However, further studies are needed for clinical verification (Table 2).

### 5.6 Hypocalcemia

Hypocalcemia (with or without symptoms) is one of the most common adverse effects of RCA, with incidence rates of 1.5–9% among patients undergoing TPE (51). The incidence of hypocalcemia is high due to the high clearance rate in the cTPE mode. Coirier et al. (33) revealed that the incidence of hypocalcemia during RCA-cTPE was 17.8–21.4% and that the incidence of hypocalcemia was higher when the replacement fluid was plasma. Hypocalcemia was classified according to Lee’s classification (37), and it was mainly grade I asymptomatic (73%) (33). Therefore, prophylactic calcium pump therapy and continuous iCa monitoring have been recommended for patients undergoing RCA-TPE (52). The risk of symptomatic hypocalcemia can be mitigated by supplementing calcium gluconate or calcium chloride (53). Numbness around the mouth and fingers, metallic taste in the mouth, nausea, chills, muscle cramps, and prolonged QT interval on the electrocardiogram are indicators of hypocalcemia. Communication disorders may lead to serious complications, such as hand-foot convulsions, arrhythmias, and seizures in patients with consciousness disorder, patients undergoing sedation and analgesia treatment, and pediatric patients. Therefore, iCa levels must be carefully monitored in these patients (51) (Table 2).

### 5.7 Other electrolyte imbalance

During RCA-TPE, the chelation of citrate with iCa increases intracellular sodium (iNa) levels in the body (1 mmol of 4% sodium citrate solution contains 3 mmol of iNa). However, it is reported that the occurrence of hypernatremia is rare after RCA-TPE (35). Citrate can also chelate magnesium. The removal of the citrate-magnesium complex by an extracorporeal circuit increases the risk of hypomagnesemia (40). However, hypomagnesemia cannot be detected using blood gas analysis. Thus, necessity of daily monitoring of magnesium levels in patients after TPE is debatable. An international survey of pediatric patients undergoing TPE revealed that 19% of respondents used magnesium supplements (38). However, magnesium supplementation is considered unnecessary for TPE (43).

## 6 Anticoagulation options for special patients

### 6.1 Anticoagulation regimen for patients at high risk of bleeding

The selection of the anticoagulation mode is challenging for critically ill patients undergoing TPE, especially for those who are at a high risk of bleeding due to surgery/trauma, active bleeding, and coagulation disorders. RCA, non-anticoagulation, and NM anticoagulation regimens have been used in patients at high risk of bleeding undergoing mTPE (7, 9, 25, 35). For critically ill patients with a high risk of bleeding, anticoagulation programs are selected based on the assessment of the patients’ coagulation function and bleeding risk, as well as anticoagulation indications and contraindications. HA and RCA are the two most widely used anticoagulant regimens in clinical practice. The use of HA or RCA in patients at high risk of bleeding undergoing TPE is controversial. Jiao et al. (35) revealed that the therapeutic effects of HA-TPE and RCA-TPE were similar, with no significant differences in the incidence of blood clotting during extracorporeal circulation. However, compared with HA, RCA can effectively reduce the risk of bleeding. Additionally, the blood transfusion requirement in the HA group was significantly higher than that in the RCA group, which may also be related to the increased number of bleeding events. Yuan et al. (7) demonstrated that the platelet and coagulation functions were not significantly different between RCA and non-anticoagulant groups among patients with bleeding tendencies. However, the incidence of pipeline coagulation in the non-anticoagulant group was higher than that in the RCA group. RCA is an effective and safe anticoagulation method for patients at high risk of bleeding. However, compared with HA-mTPE, RCA-mTPE has a higher risk of metabolic alkalosis and hypocalcemia during treatment, which requires careful monitoring and timely adjustment (35). Previous studies have reported the safety and effectiveness of NM anticoagulation in plasma exchange therapy in patients at high risk of bleeding (26). Clinicians can apply NM anticoagulation in plasma exchange therapy for patients with a high risk of bleeding.

### 6.2 Anticoagulation regimen for patients with liver failure

TPE has been used for the treatment of liver failure (54, 55). The EASL (56) Clinical Practical Guidelines on the management of acute (fulminant) liver failure cite high-volume TPE as the first-line treatment for acute liver failure. However, the use of anticoagulants for TPE in patients with liver failure is controversial because citrate is mainly metabolized by the liver. In patients with liver failure, impaired liver metabolism may lead to an increase in risk of CA, which is often exacerbated by renal impairment (57). Compared with RCA, HA can aggravate coagulation disorders in patients with liver failure, thereby increasing the risk of bleeding. Yuan et al. (7) reported that the incidence of bleeding in patients with liver failure undergoing HA-mTPE was 13.7–26.9%. Yuan et al. (42) suggested that non-anticoagulation therapy could reduce the risk of bleeding in patients with liver failure and that citrate in the plasma could meet the demand for anticoagulation. Therefore, the self-coagulation function and bleeding risk of the patients must be evaluated to select the appropriate anticoagulation regimen according to indications and contraindications. Recent studies have confirmed that patients with liver failure can metabolize citrate. Citrate used in CRRT has been confirmed to be safe for patients with liver failure (39, 58–60). Therefore, citrate is suggested as the first choice of anticoagulation for TPE in patients with liver failure (38, 57). Ma et al. (39) demonstrated that although the incidence of CA in patients with liver failure at 1 h post-TPE was 29.6%, citrate returned to physiological levels the next morning. Additionally, citrate was well tolerated without causing other discomforts. The common complications of RCA-TPE in patients with liver failure are hypocalcemia and metabolic acidosis (39). Therefore, RCA-TPE is recommended for patients with liver failure. However, the occurrence of metabolic acidosis and hypocalcemia in these patients must be monitored. Additionally, NM anticoagulation can be used for patients with contraindications to citrate, such as those with severe liver failure (13).

### 6.3 Anticoagulation regimen for patients receiving anticoagulant therapy

Inpatients or outpatients may receive anticoagulant therapy because of an underlying disease. For example, patients may receive UFH or LMWH to prevent venous thromboembolism and warfarin or direct oral anticoagulant (DOAC) for atrial fibrillation (43, 61). The use of anticoagulants for TPE in patients already receiving anticoagulant therapy varies among different centers. TPE may be performed after anticoagulants are no longer at therapeutic levels or have been reversed by antagonists (43, 61). When heparin and LMWH are used, the duration of TPE can be determined according to the half-life of the drug (62, 63). This is challenging for patients receiving warfarin or DOAC because these drugs has a long half-life, the availability of reversing agents is low, and the ability to easily monitor bleeding risk is poor. In individuals treated with warfarin, albumin is recommended as a replacement fluid. Alternatively, the anticoagulant medication can be switched to UFH or LMWH before treatment to control the anticoagulant effect (61, 64). Previous studies have reported (61–64) that TPE can be performed with citrate and heparin following standard protocols in patients who have received previous anticoagulant therapy. However, citrate and heparin increase the risk of bleeding complications in these patients.

## 7 Conclusion

Clinical TPE is mainly divided into cTPE and mTPE modes. RCA is recommended as the preferred anticoagulation method for both modes. For patients at high bleeding risk or with diseases such as liver failure, clinical studies have supported the use of RCA. However, the dosage of citrate must be strictly monitored because of the low clearance rate of citrate in the mTPE mode. Additionally, the incidence of CA and other related complications is relatively high. The most common complications of TPE using citrate anticoagulation are metabolic alkalosis and hypocalcemia. Although the symptoms are not serious, arterial blood gas, acid-base balance, and electrolyte levels must be monitored when citrate anticoagulation is applied. The occurrence of severe hypocalcemia can be clinically prevented through prophylactic calcium supplementation.
